# Nu–Gr correlation for laminar natural convection heat transfer from a sphere submitted to a constant heat flux surface

**DOI:** 10.1038/s41598-024-67382-2

**Published:** 2024-07-17

**Authors:** Qi Zhen, Tana, Yunfeng Sun, Caixia Yan, Hongzhi Wang

**Affiliations:** 1 College of Mechanical and Electrical Engineerging, Inner Mongolia Agricultural University, Saihan District, Hohhot, China; 2 College of Energy and Transpartation Engineerging, Inner Mongolia Agricultural University, Saihan District, Hohhot, China

**Keywords:** Natural convection, Nusselt number, Sphere, Constant heat flux, Correlating equation, Engineering, Mathematics and computing, Fluid dynamics, Statistical physics, thermodynamics and nonlinear dynamics

## Abstract

The work numerically investigated laminar natural convection heat transfer from the single sphere with a constant heat flux surface in air over the wide range of Grashof number ($10 \le Gr \le 10^{7}$). The more efficient and precise numerical method based on Bejan et al. was employed here, the accuracy of which has been confirmed through validation against a single sphere case. The heat transfer characteristics were systemically analyzed in terms of isothermal contours and streamlines around the sphere, dimensionless temperature and velocity profiles. Additionally, local Nusselt number as well as local pressure and friction drag coefficients were studied with different Grashof number. In comparison to the sphere with uniform heat flux surface, the heat transfer from the isothermal sphere was found to be enhanced attributable to a more robust buoyancy force and a steeper temperature gradient. Moreover, the average Nusselt number for the sphere with a constant heat flux between 60.4 and 98.6% of the isothermal sphere’s value, this range being contingent upon the specific Grashof number. What’s more, the proposed correlation addresses a notable void in the predictive understanding of heat transfer from the sphere with uniform heat flux, which is scenario prevalent in various engineering applications, particularly for the cooling of electrical and nuclear systems, and offer values for academic research.

## Introduction

Natural convection is propelled by the density differential induced by the temperature gradient within a fluid. This mechanism offers an economical, low-noise and convenient way to cool a heated wall of an adjoining fluid, and has thus been frequently employed in thermal energy storage systems and pebble bed reactors in recent years^1^. The phenomenon of heat transfer via external natural convection over a single sphere submerged in a quiescent fluid is a classical issue in this field, and it is frequently utilized in nuclear power engineering^2^. Illustrative instances include fuel spheres in pebble bed and high-temperature gas-cooled reactor^3^ and ADS granular spallation target^4^. As Bejan^5^ observed, when the heat exchange on a sphere’s surface is derived from radiation heating, a boundary condition of the constant heat flux is applied. With the burgeoning development of nuclear energy applications, research on spherical surfaces subjected to constant heat flux is becoming increasingly essential. However, there has been a paucity of studies investigating natural convection heat transfer over spheres with a constant heat flux surface in this field.

In contrast, most researchers have concentrated on investigating the flow and heat transfer characteristics of an isothermal sphere, owing to its extensive practical relevance. In early analytical works, the main concerns were the characteristics of flow and heat transfer at a high Grashof number over a sphere with a constant temperature surface by using boundary layer assumptions. Merk et al.^6–8^ are often considered as pioneers in the development of solutions for fluid flow through a vertical cone, specifically applicating the axisymmetric form. Chiang et al.^9^ first investigated the precise analysis of laminar free convection in spheres by considering the specified surface temperature and surface heat flux. Jafarpur et al.^10^ created a simple and accurate approximation of the analytical method to solve the area-averaged Nusselt number of free convective heat transfer from an isothermal sphere in a specific range of Rayleigh numbers and in all Plante number ranges. Potter et al.^11^ researched the effect of large Grashof numbers on free convective heat transfer along the thermal rod in the case of reduced gravity. Furthermore, the limiting case at very low Grashof number was implemented by using asymptotic expansion techniques^12–14^. Fendell^12^ studied a very small heated isothermal sphere introduced into a fluid in hydrostatic equilibrium at low Reynolds and Grashof numbers. Hossain et al.^13^ analyzed the natural convection and heat transfer of laminar flow around an isothermal sphere for the Grashof number range between zero and unity and for Prandtl number around unity, using the asymptotic unfolding technique. Singh et al.^14^ solved A numerical model for the free convective flow about an isothermally heated sphere by the series truncation method, with consideration given to a small values of the Grashof number (0.01–0.1).

The first numerical solution for the heat transfer of a sphere by natural convection in air was provided by Geoola and Cornish^15^. They presented the local and overall Nusselt number and drag coefficient by applying the full Navier–Stokes and energy equations for $0.05\le Gr\le 50$ and $Pr=0.72$. Moreover, isothermal contours and streamline patterns were also reported. In their follow-up analysis^16^, they solved the transient heat transfer for Grashof number up to 12,500 and Prandtl number of 0.72, 10 and 100. Jia and Gogos^17,18^ analyzed the transient and steady-state regimes of free convection heat transfer over a heated sphere for a wide range of Grashof number $10\le Gr\le {10}^{8}$ and two values of the Prandtl number, namely, $Pr=0.72$ and $7.0$. Yang et al.^19^ carried out transient laminar natural convection from an isothermal sphere in the range of ${10}^{5}\le Gr\le {10}^{9}$ and a wide range of Prandtl number ($Pr=0.02, 0.7, 7\text{ and }100$). A plume such as a mushroom cap forming above the sphere and rising upward continuously with time was observed, and the average Nusselt number and the total drag coefficient for the range of Grashof and Prandtl number studied in the steady state were determined. Based upon the combination of theoretical, numerical and experimental studies, correlation equations for the heat transfer coefficient were given by Churchill et al.^20,21^ for natural convection over spheres in stagnant fluids. Jafarpur and Yovanovich^10^ also proposed a correlation for the dependence of the average Nusselt number on the Rayleigh number in the range $0\le Ra\le {10}^{8}$ and all Prandtl number for laminar free convective heat transfer from a single isothermal sphere. The results of their work are in accordance with other empirical formulas and experimental data. The preceding works are restricted to a single sphere for natural convection heat transfer in unlimited and static Newtonian fluid. Recently, Wu et al.^22^ investigated the forced convection heat transfer characteristics of an endothermic biomass sphere in supercritical water (SCW), showing the local heat transfer distribution on the surface of the particle; the enhancement at the front of the particle is significantly greater than that at the rear. Additionally, the study suggests a new correlation for forced convection heat transfer applicable to SCW flow around an endothermic sphere. Prhashanna and Chhabra^23^ described the free convection heat transfer from a heated sphere immersed in quiescent power-law fluids for the following ranges of conditions: $10\le Gr\le {10}^{7}; 0.72\le Pr\le 100$ and $0.4\le n\le 1.8$. They concluded that the shear-thinning behavior can enhance the heat transfer rate by up to threefold, whereas shear thickening can impede it up to 30% ~ 40% with respect to that in Newtonian fluids.

The study of natural convection over a sphere with a constant heat flux surface originated from Chiang et al.^9^. Later, a theoretical analysis of steady magnetohydrodynamic (MHD) natural convective flow and mass transfer through a porous medium bounded by an infinite vertical porous plate with constant heat flux was performed by Raptis and Kafoussias^24^. Shafiq et al.^25–27^ investigated heat transfer phenomena in both magnetic fluids and nanofluids based on the numerical simulations. The study involved the analysis of parameters such as Brownian motion, thermophoresis, and Newtonian heating, as well as the numerical analysis of Sherwood number, Nusselt number, and surface friction coefficient. These studies above provided guidance for potential manufacture of devices. In latest study^28^, they studied the heat transmission performance of Darcy–Forchheimer tangent hyperbolic radiative inclined cylindrical film movement in parabolic trough solar collector with an irregular heat sink/source. The outcomes proved the fluids featuring tangent hyperbolic rheological conductivity were obligatory for active rate of heat diffusion which be employed in Parabolic Trough Solar Collector. Huang and Chen^29^ studied the effects of the Prandtl number and mass transfer from a sphere with blowing and suction, where the sphere surface is set to a nonuniform temperature or heat flux. The transient laminar natural convection flow over a sphere subjected to a constant heat flux for high Grashof number (${10}^{5}\le Gr\le {10}^{9}$) and a wide range of Prandtl number ($Pr=0.02, 0.7, 7$ and $100$) was examined by Saito et al.^30^. They noted that the hydrodynamic boundary layer thickness is greater than the thermal boundary layer thickness for all Prandtl number. Finally, they also correlated the overall Nusselt number as a function of $Gr$ and $Pr$. Yan et al.^31^ considered unsteady free convection flow from a sphere in a porous medium compared with step changes in surface temperature or heat flux. The solutions of the transient boundary layer for both small and large Rayleigh number are obtained using a classical finite difference method. Gokulavani et al.^32^ analyzed the effect of double-diffusive convection in a hybrid nanofluid filled in an inclined open cavity with heat generation/ absorption. The results reported that an inclination angle suppresses the heat and mass transfer rate in an open enclosure. Navaneethakrishnan et al.^33^ investigated the flow structure and heat transfer characteristics within a vented channel-driven cavity. The results showed that higher Reynolds and Richardson numbers enhance heat transfer, but an increased Hartmann number reduces it.

As described above, in both fundamental academic research and industrial applications, a single sphere with constant heat flux heat transfer plays an important role in laminar natural convection. However, most of the previous work focused only on natural convection over a single sphere over a wide range of Grashof number or Prandtl number to investigate the characteristics of the flow and heat transfer in a large space. They proposed correlating equations for calculating the average Nusselt number as a function of the Grashof number or both the Grashof number and Prandtl number, and the correlating equations accurately predicted the heat transfer coefficient to satisfy the needs of scientific experiments and engineering calculations. Despite the analyses of natural convection over a sphere subjected to a constant heat flux, the correlation equations are given. However, its application scope is too small (only ${10}^{5}\le Gr\le {10}^{9}$), and the addition of the *Pr* number sacrifices the accuracy of the formula in air.

Based on the discussion, there is still a lack of available results concerning natural convection heat transfer for a sphere with subjected to a constant heat flux within a wide range of Grashof numbers. For multi-spheres heat transfer in the engineering applications, like the pebble bed in the high-temperature gas cooled reactors, the storage industry of the spherical fruit and vegetable and ADS granular flow target, the empirical formula usually requires a larger *Gr* number range and computational accuracy. Hence, in this paper, a comprehensive investigation of the heat transfer and flow characteristics of a single sphere with uniform heat flux surface in air by natural convection. Furthermore, a more economical numerical model proposed by Bejan et al.^28^ was used in this work. The reported results herein consist of isothermal contours and streamline distributions in the vicinity of the sphere, temperature and velocity profiles, local and average Nusselt number, and comparisons with those of the isothermal sphere. A detailed analysis of all obtained results has been conducted, as expected, a correlation equation for predicting accurately the heat transfer rate for a sphere cooled by natural convection in air have been presented with the range of $10\le Gr\le {10}^{7}$.

## Numerical methodology and mathematical description

**Figure 1:**
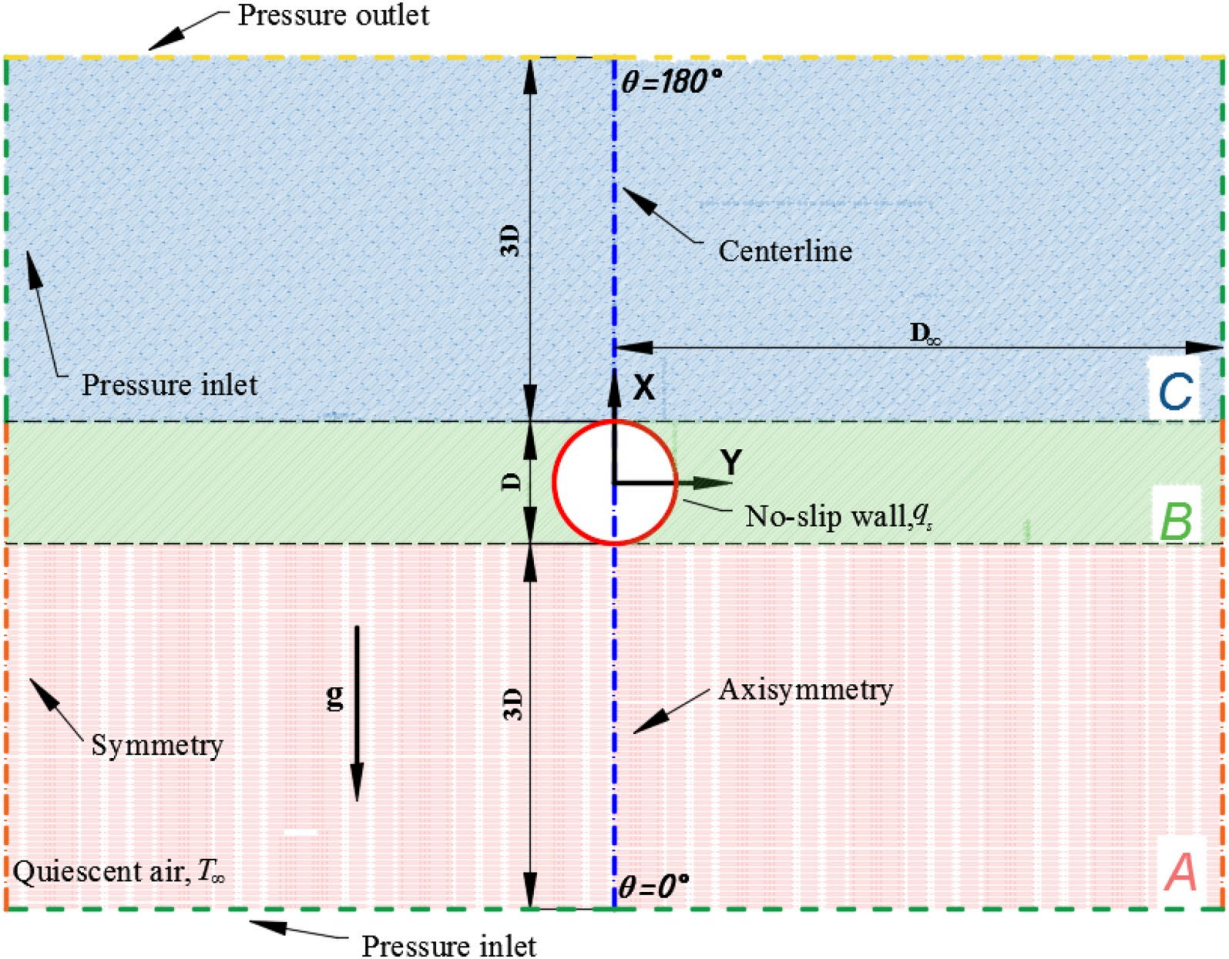
Schematic representation of the flow and the computational domain.

In this study, the external natural convection heat transfer over a single sphere is examined with its surface set to maintain a constant heat flux rate at a uniform value, ${q}_{w}$. This sphere is immersed in a region filled with quiescent air, which is also maintained at a constant temperature and pressure of ${T}_{\infty }$ and ${p}_{\infty }$, respectively. Based on the constant heat flux condition, the surface of the sphere obtains a higher temperature ${T}_{w}$ than that of the fluid, as shown schematically in Fig. 1. Moreover, the temperature gradient existing in the vicinity of the sphere owing to the effect of thermal expansion leads to the appearance of a density gradient. Hence, an upward axisymmetric flow induced by the buoyancy emerges around the sphere. As previously described, the fluid is assumed to be in laminar and steady-state conditions within the range of the Grashof number between $10$ and $10^{7}$, and the flow remains axisymmetric around the sphere. Consequently, it is feasible to use the two-dimensional model to simulate the three-dimensional case using the half-domain of the structure in the present study.

**Figure 2:**
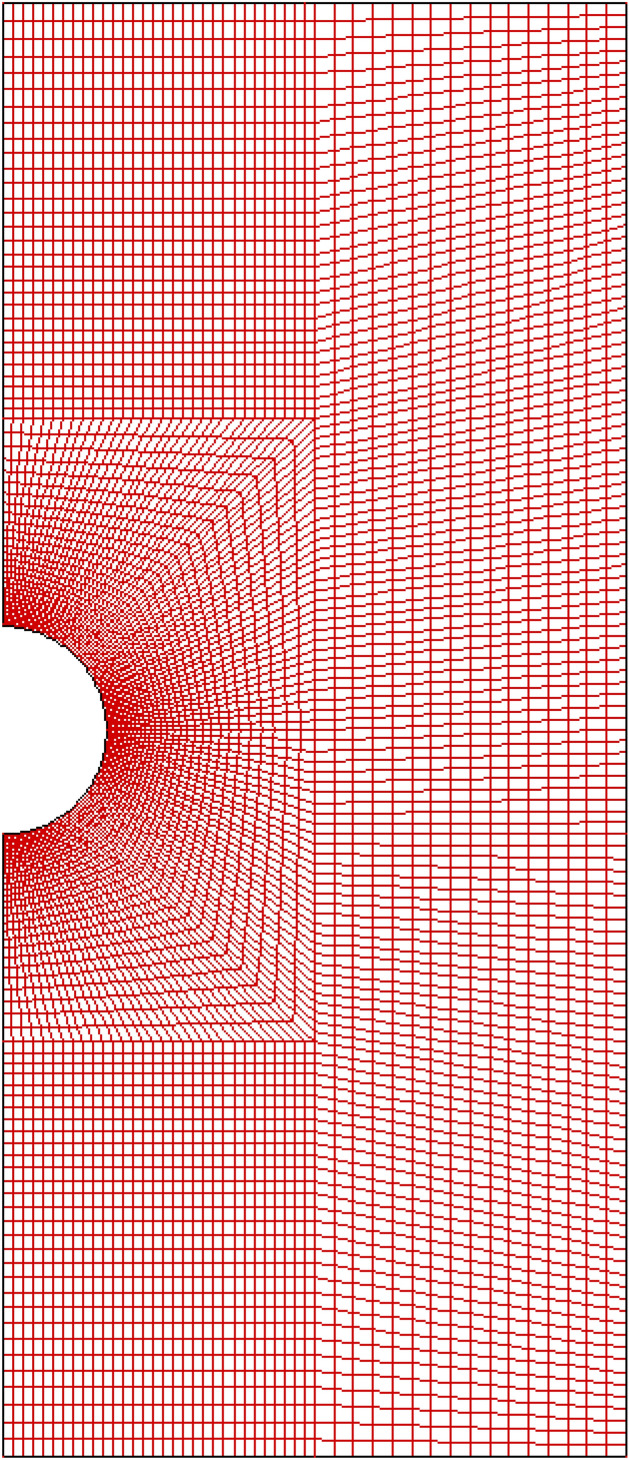
Schematic representation of the computational grid with a closer view of the grid around the sphere.

The laminar natural convection heat transfer from a sphere, which is modeled as shown schematically in Fig. 1, is analyzed. However, the study of natural convection heat transfer usually requires a large space to avoid impacting the flow and heat transfer from the concerned sphere. In the prior studies, the conventional practice for the spheres is to be immersed in a large sufficiently enclosure, the ratio of inner to outer boundary D_∞_/D is about 8, 12.5, 25, 80^15,16,23,34^ and such a model would require a large computational domain according to the above analytical works. It is foreseeable that such enormous computational effort results from the large number of grids. However, the excellent and economical numerical model proposed by Bejan et al.^35^ for natural convection has been employed in this work that the scale of the computational domain is reduced to 3D. According to Bejan et al.^35^, the computational domain is primarily divided into three sections, the inlet section, the actual channel section, and the outlet section. To avoid artificial acceleration of the fluid flowing through the vertical boundary of the outlet section due to the presence of updrafts or chimney effects, an inlet boundary condition is set on one side of the outlet section, especially away from the topmost cylinder, which is also the main difference from the traditional model. The reliability and accuracy of the numerical model was tested by comparing the results of a single tube at Ra = 10^3^ and Ra = 10^4^ and the theoretical and experimental results of a bundle of tubes in air. As anticipated, the results are in fair agreement with a deviation of only around 6%. More details on the accuracy of Bejan’s numerical model have been reported in previous articles^36–38^, and thus, this discussion is not repeated here. In the present case, Fig. 2 shows a schematic representation of the uniform calculated grid around a single sphere. It should be noted here that the temperature and velocity gradients are exceptionally large in the vicinity of the sphere, but far from the sphere, all of them become moderate. Therefore, the computational domain is divided into two subdomains that have different grid densities. A fine grid is required near the sphere surface to capture the dramatic variation in the velocity and temperature distributions, while a relatively coarse grid is used in the remaining region. There are three sections shown in Fig. 1, consisting of an inlet section, the concerned spheres section and an outlet section, which are compatible with regions A, B, and C in the domain, respectively. The origin of the Cartesian frame $\left( {x, y} \right)$ is located at the center of the sphere. Gravity acts in the negative $x$-direction. Moreover, the fluid is assumed to be incompressible, and the effects of bulk viscous dissipation and radiation are negligible. Under these conditions, the control equations of continuity, momentum and energy are simplified as shown in Table 1, where $\vec{u}$ is the velocity vector, $\rho$ is the density, $p_{s}$ is the gauge pressure relative to the far-field pressure, $u$, $v$ and $\alpha$ are the $x$ and $y$ direction velocities and thermal diffusivity components, respectively, and $\beta$ is the coefficient of thermal volumetric expansion. The thermophysical properties are assumed to be independent of temperature, but the density varies with temperature, as derived from the Boussinesq approximation, $\left( {\rho_{\infty } - \rho } \right) = \rho \beta \left( {T - T_{\infty } } \right)$. Due to the small temperature difference, the $\beta$ can be considered by $\beta = 2/\left( {T_{w} + T_{\infty } } \right) = 1/T_{m}$ in line with the ideal gas model^5^.

**Table 1:** The governing equations with constant properties.

Definitions	Formula	No
Continuity equations	$\nabla \cdot \vec{u} = 0$	(1)
Momentum equations of $x$-direction	$u\frac{\partial v}{{\partial x}} + v\frac{\partial v}{{\partial y}} = - \frac{1}{\rho }\frac{{\partial p_{s} }}{\partial y} + \vartheta \nabla^{2} v + \rho g\left( {T - T_{\infty } } \right)$	(2)
Momentum equations of $y$-direction	$u\frac{\partial u}{{\partial x}} + v\frac{\partial u}{{\partial y}} = - \frac{1}{\rho }\frac{{\partial p_{s} }}{\partial x} + \vartheta \nabla^{2} u$	(3)
Energy equation	$u\frac{\partial T}{{\partial x}} + v\frac{\partial T}{{\partial y}} = \alpha \nabla^{2} T$	(4)

where $\nabla^{2} = \partial^{2} /\partial x^{2} + \partial^{2} /\partial y^{2}$; $u$, $v$ are the x and y direction dimensionless velocities, $p$ is the gauge pressure; $\vartheta$ and $\alpha$ are the kinematic viscosity and the thermal diffusive coefficient, respectively.

The setting of the boundary conditions is also shown in Fig. 1. The bottom side of the computational domain is regarded as the ‘inlet’, where the fluid temperature is set at the ambient temperature, $T_{\infty }$. Simultaneously, the top side represents the ‘outlet’, whose total backflow temperature is also set to $T_{\infty }$. It is defined as a no-slip wall boundary condition and a specified constant heat flux at the sphere surface. In addition, due to the axisymmetry of the flow, the present work is only carried out in the right half of the domain, i.e., $X \ge 0$. As a result, the centerline of the domain should be set as an axisymmetric boundary condition. On the other hand, the right sides of the inlet section and the sphere section are also defined as symmetric boundary conditions. The effect of the flow characteristic becomes negligible when the right side of the calculation domain is distant from the concerned sphere. Therefore, it is reasonable that symmetrical boundary conditions are applied to these surfaces, which can significantly reduce the domain size compared to that of conventional methods. The following boundary conditions are applied in Table 2. To normalize all variables appearing in the results, the dimensionless variables are defined by using the scaling variables presented as Eq. (10) in Table 3.

**Table 2:** The boundary conditions.

Definitions	Formula	No
Sphere surfaces	$u = v = 0,p_{s} = 0, q = q_{s}$	(5)
Inlet boundary	$p_{0} = 0, \partial u/\partial x = 0,v = 0,T = T_{\infty }$	(6)
Outlet boundary	$p_{0} = 0, \partial u/\partial x = 0, v = 0, T = T_{\infty }$	(7)
Axis of symmetry	$\partial u/\partial y = 0, v = 0, \partial T/\partial y = 0$	(8)

**Table 3:** Parameter definitions and formulas.

Nomenclature	Formula	No
Thermal expansion coefficient	$\beta = - 1/\rho \left( {\partial \rho /\partial T} \right)_{p}$	(9)
Dimensionless variables	$X = \frac{x}{R}, Y = \frac{y}{R},T^{*} = \frac{{T - T_{\infty } }}{{q_{s} R/k}}$ $p_{s}^{*} = \frac{{R^{2} }}{{\rho \vartheta^{2} Gr}}p_{s} , U = \frac{{RGr^{1/2} }}{\vartheta }u , V = \frac{{RGr^{1/2} }}{\vartheta }v$	(10)
Local Nusselt number	$Nu_{\theta } = h_{\theta } R/k = - \left( {\partial T^{*} /\partial n_{s} } \right)_{surface}$	(11)
Average Nusselt number	$\overline{Nu} = \overline{h}R/k = 1/S\smallint Nu_{\theta } dS$	(12)
Grashof number	$Gr = \frac{{g\beta q_{s} R^{4} }}{{k\vartheta^{2} }}$	(13)
Prandtl number	$Pr = \frac{\vartheta }{\alpha } = \frac{{c_{p} \mu }}{k}$	(14)
Pressure drag coefficient	$C_{D,p} = \left( {F_{D,p} } \right)/(\frac{1}{2}\rho U_{c}^{2} \pi R^{2} ) = \smallint C_{p} n_{x} dS$	(15)
Viscous drag coefficient	$C_{D,\mu } = \left( {F_{D,f} } \right)/(\frac{1}{2}\rho U_{c}^{2} \pi R^{2} ) = \frac{2}{{\sqrt {Gr} }}\left( {\frac{{\partial U_{\theta } }}{\partial r}} \right)_{wall} = \smallint 4\tau_{w} n_{s} dS$	(16)
Total drag coefficient	$C_{D} = C_{D,p} + C_{D,\mu }$	(17)
Pressure coefficient	$C_{p} = \left( {p_{s} - p_{0} } \right)/\left( {\frac{1}{2}\rho U_{c}^{2} } \right)$	(18)
Reference velocity	$U_{c} = \sqrt {\left( {g\beta R^{2} q_{s} } \right)/\left( {2k} \right)}$	(19)

where $R$ is the radius of the sphere; $k$ is the thermal conductivity; $n_{x}$ is the outward drawn unit vector in the x direction, $n_{s}$ is the outward drawn unit normal vector on the surface of the sphere; $F_{D,f}$ is the local wall shear stress, $p_{s}$ and $p_{0}$ are the local gauge pressure and the ambient pressure, respectively.

The governing Eqs. (1), (2), (3), and (4)^29,32,37^ are integrated by using a control-volume formulation and discretized by the finite difference method (FDM). The results are solved iteratively by employing the boundary conditions [Eqs. (5)–(8)] to the solver of ANSYS Fluent 15.0^36–38^. The convection terms in the momentum and energy equations are discretized through quadratic upstream interpolation for the convective kinematics (QUICK) scheme. The semi-implicit method for pressure-linked equations (SIMPLE) algorithm is utilized to handle pressure–velocity coupling. The convergence criteria of 10^–6^ for the continuity, momentum and energy equations were specified.

## Results and discussion

Numerical simulations are conducted over the range of the Grashof number from $10$ to $10^{7}$ and for ${ }Pr = 0.72$. To clearly understand some dimensionless parameters that determine mainly the characteristics of natural convection heat transfer, a major definition of these dimensionless parameters should be provided for Eq. (10), which adopted a specific scales as follows: the process of non-dimensionalization is performed based on the radius of the sphere, the thermal diffusivity of the air, and the temperature difference within the system^33,36^.

The Nusselt number ($Nu$) is a dimensionless parameter defined as the ratio of the convective to conductive heat transfer resistance from the laminar boundary layer of the fluid. It represents a criterion for the strength of the convective heat transfer. The local and average Nusselt number $Nu_{\theta }$ and $\overline{Nu}$ of the sphere are calculated, and their definitions are presented in Eqs. (11) and (12), respectively^37^.

The Grashof number ($Gr$) reflects the relative relationship between the buoyancy and viscous force caused by the temperature difference of each part of the fluid. It is a key parameter for measuring the intensity of natural convective heat transfer. However, the Grashof number is different from the definition of a surface with a constant temperature, as shown in Eq. (13) in Table 3, which is based on the sphere radius and heat flux of the surface. Equation (13) shows that the larger the Grashof number is, the stronger the flow.

The Prandtl number ($Pr$) represents the ratio of the momentum diffusing thickness to the heat spreading thickness, which is determined by the effect of the physical properties of the fluid on the convective heat transfer process. The definition formula is given as Eq. (14) in Table 3.

Moreover, the governing equations and boundary conditions usually indicate that the temperature and flow region are affected by the Grashof number and Prandtl number. In this work, the fluid is air, and $Pr$ is fixed at 0.72.

The drag coefficient ($C_{D}$) is used to quantify the drag of an object in a fluid environment, and the shearing and normal forces are given rise to buoyancy-driven flow around the sphere, thereby resulting in a drag force acting on the sphere in the $x$ direction. The drag force is usually termed the drag coefficient ($C_{D}$), which is decomposed into two components, the friction drag coefficient ($C_{D,\mu }$) and the pressure drag coefficient ($C_{D,p}$), resulting from the influence of the viscosity and the curved surface of the sphere, respectively^36,37^. These are defined as Eqs. (15), (16), and (17) in Table 3. In addition, the pressure coefficient ($C_{p}$) and reference velocity ($U_{c}$) are defined as Eqs. (18) and (19), respectively. The Nusselt number ($Nu_{\theta } ,\overline{Nu}$) and drag coefficient ($C_{D} ,C_{D,\mu } , C_{D,p}$) are related to the Grashof number, and their relationships will be investigated in the following sections^36–38^.

### Validation of results

Generally, the study of natural convection is reasonable for setting the sphere to be immersed in such a sufficiently large enclosure. The minimum ratio of $D_{\infty } /D$ is maintained at more than 80 times so that the boundary conditions can be guaranteed to have no influence^23^. Bejan^35^ proposed a new numerical model for natural convection from horizontal cylinders that reduces the computational domain by less than 10 times, thus reducing the computational cost. In contrast to cylinders obtained via plane symmetry, a sphere is acquired via the axisymmetry of the semicircle under two-dimensional conditions. This means that the new numerical method must be verified to determine its reliability and precision.

**Figure 3:**
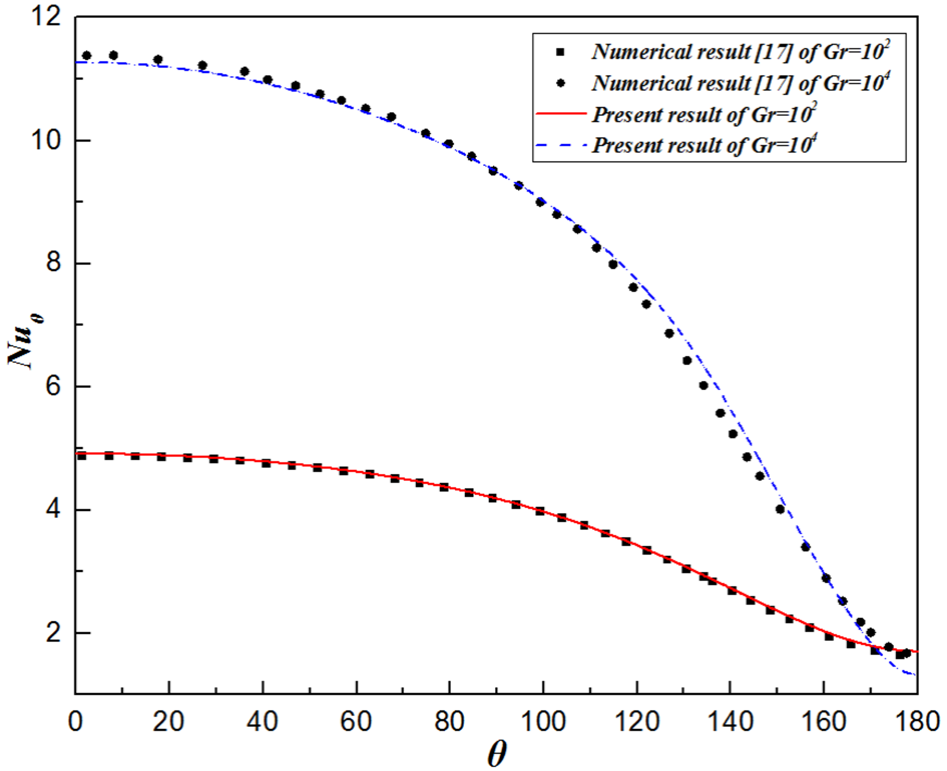
Comparison of the surface temperature profiles with previous numerical results for $\theta =0^\circ$ and $180^\circ$.

**Figure 4:**
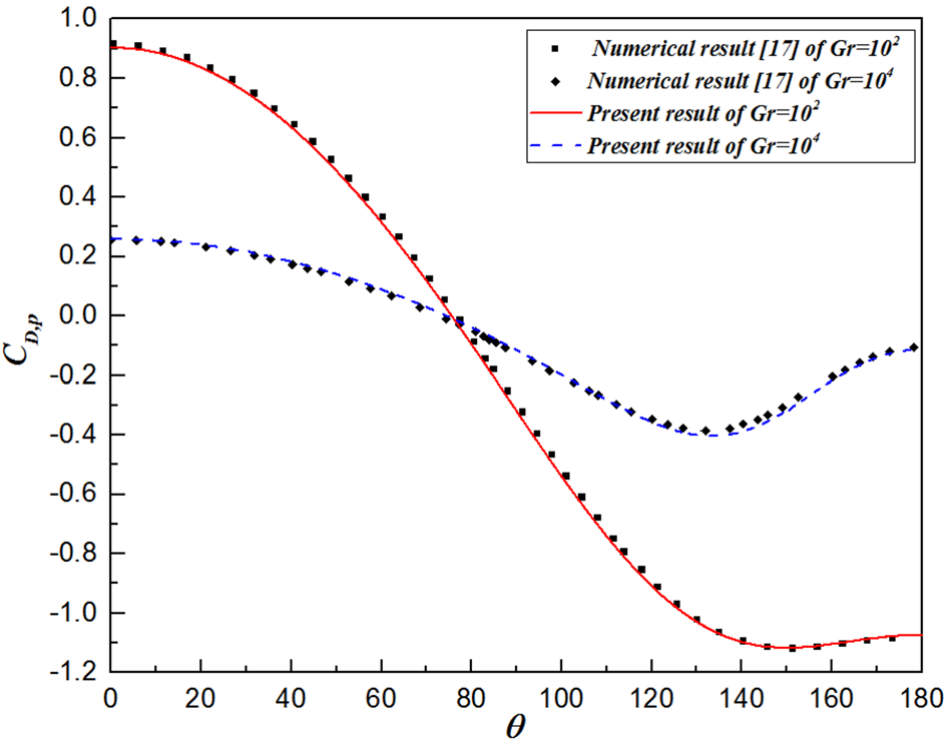
Comparison of the local pressure coefficients with previous numerical results for $\theta =0^\circ$ and $180^\circ$.

As mentioned above, many previous studies have provided numerical results for the problem of isothermal sphere in the range of Grashof number between $$10$$ and $$10^{7}$$. Table 4 shows comparisons of the pressure ($$C_{D,p}$$) and total ($$C_{D} )$$ drag coefficients, as well as the average Nusselt number ($$\overline{Nu}$$)) for the numerical results of prior literature^17,23^ and the present results. As expected, all parameters are in near-perfect agreement between the literature and present values where the maximum deviation is less than $${ } \pm 1.5{\text{\% }}$$. Moreover, to further validate the results, for $$Gr = 10^{2}$$ and $$10^{4}$$, the variations in the local Nusselt number and the local pressure drag coefficient at different angles $$\theta$$ with respect to the radial distance are also compared with those of Jia and Gogos^17^, as shown in Figs. 3 and 4, respectively. The trends of the present results are in excellent agreement. In general, the close predictions shown in Figs. 3 and 4 constitute a more rigorous test of the reliability than that shown in Table 4.

**Table 4:** Comparison of the present results with those of prior literature^17,23^.

	$C_{D,p}$	$C_{D}$	$\overline{Nu}$
$$Gr = 10$$			
Jia and Gogos et al.^17^	3.270	9.340	2.930
Prhashanna and Chhabra^23^	3.055	9.021	2.960
Present work	3.103	9.071	2.981
$$Gr = 10^{3}$$			
Jia and Gogos et al.^17^	0.800	2.030	5.705
Prhashanna and Chhabra^23^	0.811	2.053	5.668
Present work	0.800	2.041	5.650
$$Gr = 10^{5}$$			
Jia and Gogos et al.^17^	0.270	0.590	14.218
Prhashanna and Chhabra^23^	0.278	0.607	14.191
Present work	0.278	0.605	14.220
$$Gr = 10^{7}$$			
Jia and Gogos et al.^17^	0.120	0.214	41.810
Prhashanna and Chhabra^23^	0.098	0.196	41.068
Present work	0.100	0.195	41.212

The numerical result is strongly influenced by the domain of the computational zone and grid size. To eliminate these interferences, Tables 5 and 6 show the domain and grid independence tests, respectively. There is usually a thicker boundary layer and stronger viscosity effect at smaller Grashof number; hence, a larger area of the domain zone is needed. The choice of the width of the calculative domain $(D_{\infty } )$ is associated with the Grashof number, which determines the thicknesses of the boundary layers. Therefore, in the present study, two kinds of domain independence validations are carried out for the maximum and minimum value of the Grashof number, and the calculative domain $(D_{\infty } )$ is systematically varied as $10D$, $20D$ for $Gr = 10$ and $3D$, $6D$ for $Gr = 10^{7}$, as shown in Table 5. The test clearly indicates that the changes in the values of $C_{D}$, ${ }C_{D,p}$, and ${ }\overline{Nu}$ are less than 0.15%, even if the size of the computational domain is doubled. Therefore, the value of $D_{\infty }$ is fixed to ${ }10D$ for $Gr = 10{ }$ and $10^{2}$, whereas it is three times the sphere diameter for $Gr \ge 10^{3}$ to avoid wasting computational effort, and it is also confirmed that these sizes are sufficient. On the other hand, it is well known that the larger the value of the Grashof number is, the thinner the thermal and velocity boundary layers are; therefore, for higher Grashof number, a finer mesh is required to resolve the larger gradients of the velocity and temperature in the thinner boundary layers. With this consideration, two different grid independence tests are performed at $Gr = 10^{7}$, as reported in Table 6. The maximum change in case 1 is less than 0.11% compared with that in case 2, which indicates that case 1 is fine enough and requires relatively less CPU time to reach convergence; therefore, case 1 with $\delta /D = 0.01$ is chosen. In summary, it is justified that the calculative domain of $10D$ for $10 \le Gr \le 10^{2}$, $3D$ for $Gr \ge 10^{3}$ and the grid density of case 1 are satisfactory for the present work.

**Table 5:** Domain independence tests.

Domain length			$C_{D,p}$	$C_{D}$	$\overline{Nu}$
Inlet section	Outlet section	$D_{\infty }$			
$$Gr = 10$$					
$3D$	$3D$	$10D$	2.422	7.072	2.939
$6D$	$6D$	$20D$	2.417	7.075	2.934
$$Gr = 10^{7}$$					
$3D$	$3D$	$3D$	0.0145	0.0292	24.904
$6D$	$6D$	$6D$	0.0148	0.0293	24.868

**Table 6:** Grid independence tests at ${ }Gr = 10^{7}$.

Grid	Total. no. of cells	Smallest cell size $\delta /D$	$C_{D,p}$	$C_{D}$	$\overline{Nu}$
$Case 1$	980,202	0.01	0.0145	0.0292	24.904
$Case 2$	2,791,076	0.005	0.0145	0.0293	24.878

### Isothermal and streamline contours

**Figure 5:**
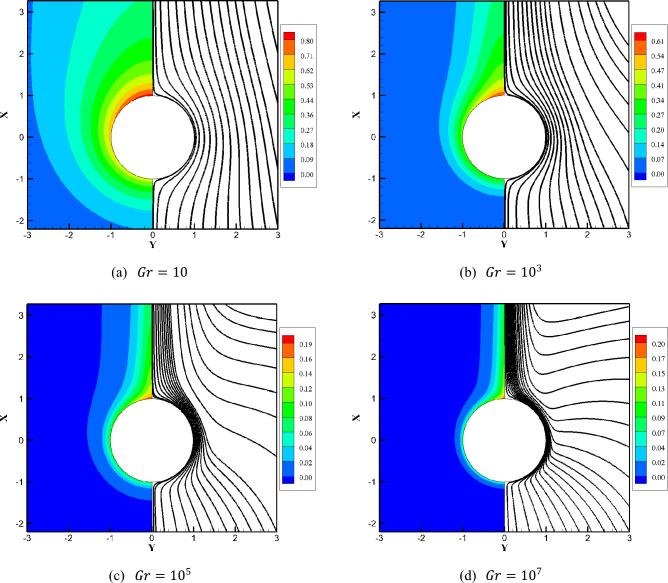
The Grashof number of the isotherm contours (left half) and streamlines (right half) in the vicinity of the sphere varied at $10\le Gr\le {10}^{7}$.

Isothermal and streamline contours are typically used to qualitatively illustrate the temperature distribution and fluid flow patterns along a sphere’s heated surface. Figure 5 displays the isothermal contours (left half) and streamline patterns (right half) around the sphere’s surface for Grashof number ranging from 10 to 10^7^. By analyzing the structural changes in the temperature and velocity field, how to influence heat transfer and flow from a constant heat flux sphere with different Grashof number can be revealed. As shown in Fig. 5, the fluid is pushed toward the heated sphere due to the density difference causing flows to ascend along the surface. A steady-state and symmetric buoyant plume is formed above the sphere, whose center is gradually transferred from the lower section (at low ${ }Gr$) toward the right-upward corner (at high ${ }Gr$) of the flow areas with increasing Grashof number. At low Grashof number, the viscous force of the fluid is so large that the flow still adheres to the surface of the sphere. This means that convection is weak due to the small buoyant force and heat transfer is dominated by conduction. It is conceivable that when $Gr$ is very small even when the fluid is at rest, heat conduction occurs solely between the sphere and fluid. When the Grashof number reaches 10^7^, the convective effect becomes more pronounced than the conductive effect. The fluid motion is guided by the stronger buoyancy, predominating over the momentum and heat transfer processes. Additionally, with higher Grashof number, both the thermal and velocity boundary layers become progressively thinner. The reason is that the buoyant force leads to heated fluid arising from the vicinity of the sphere, and the cold fluid fills from the sides and the lower part of the region. This observation from Fig. 5 explains why a larger Grashof number leads to a stronger buoyancy force. Consequently, the rate of heat transfer positively correlates with the Grashof number, and no recirculation zone or flow separation appears, even at $Gr = 10^{7}$.

### Distributions of the velocity and temperature along radial direction at various angles

**Figure 6:**
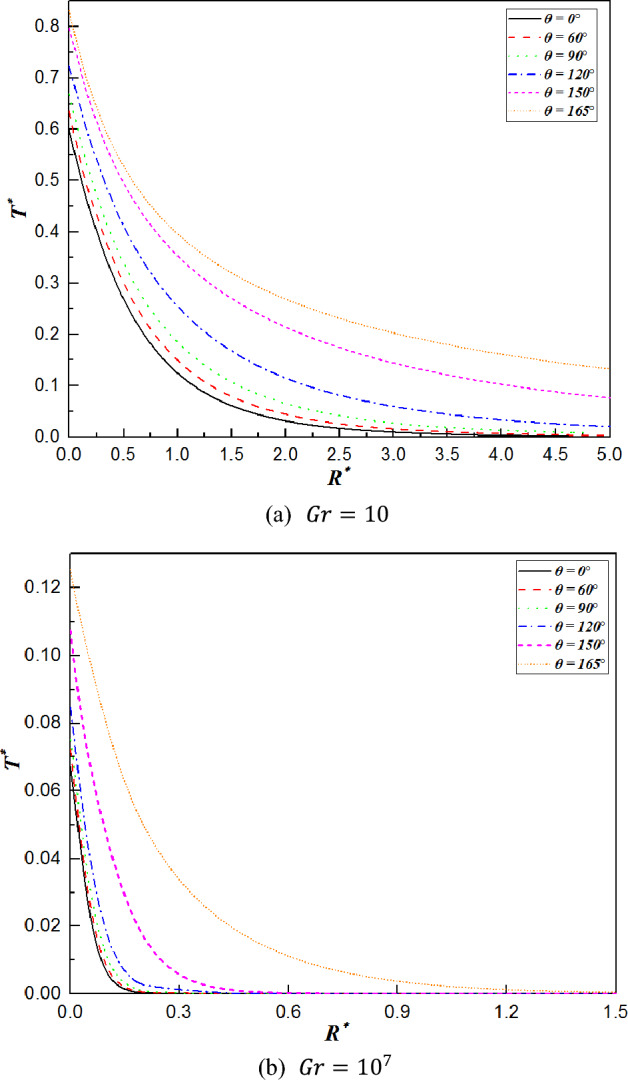
Temperature distribution along various radial directions for $Gr=10$ and $Gr={10}^{7}$.

**Figure 7:**
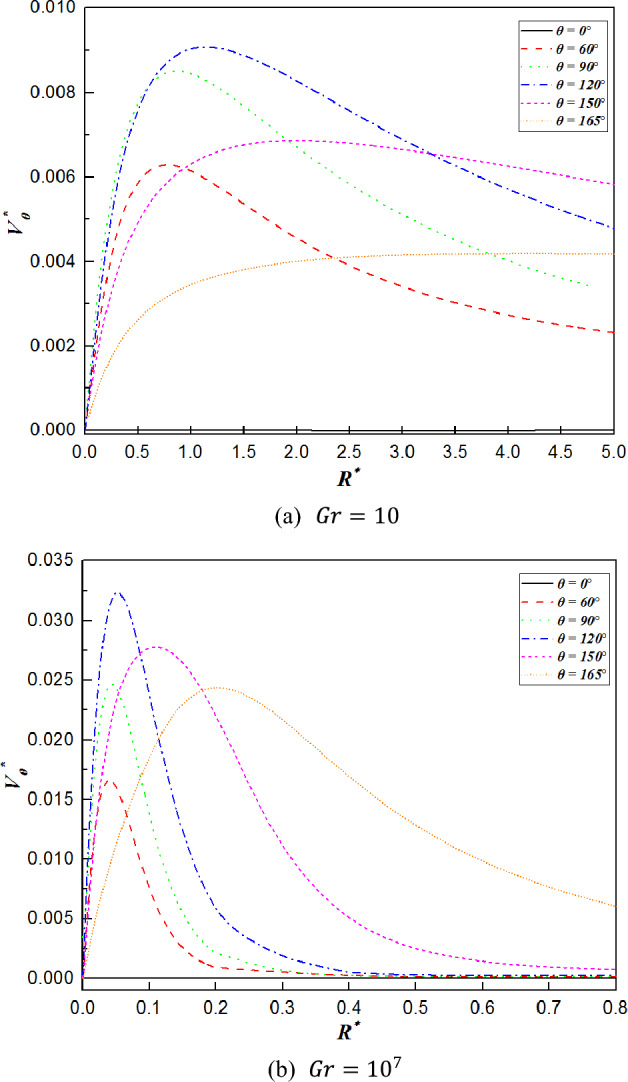
Tangential velocity distribution along various radial directions for $Gr=10$ and $Gr={10}^{7}$.

**Figure 8:**
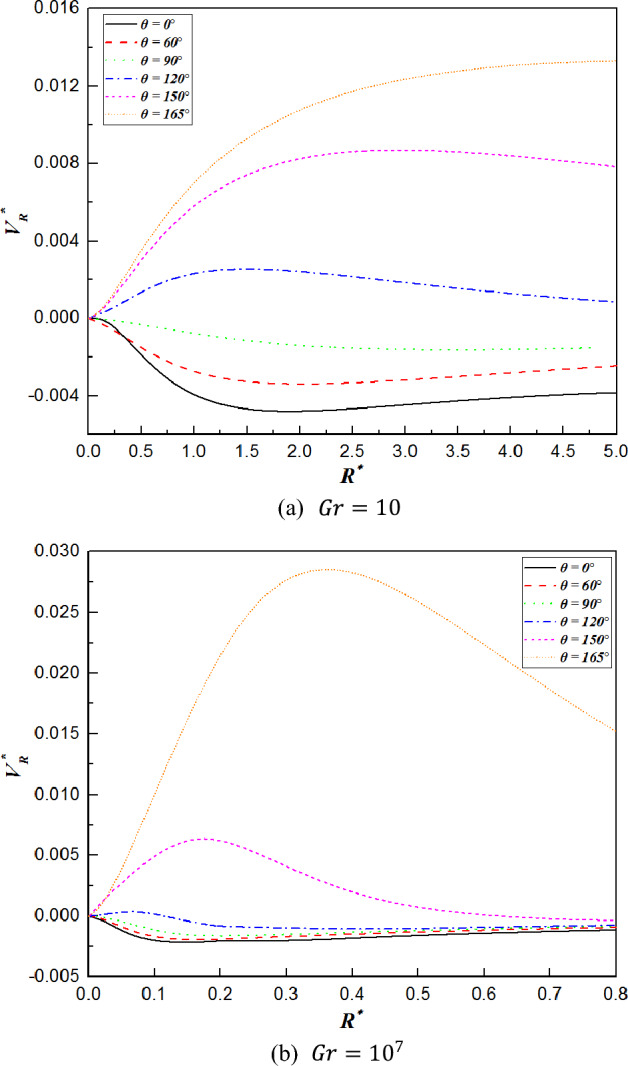
Radial velocity distribution along various radial directions for $Gr=10$ and $Gr={10}^{7}$.

The distributions of temperature and velocity along the radial direction are displayed for quantitatively analysis from Figs. 6, 7 and 8, and the polar angles (0°, 60°, 90°, 120°, 150°and 165°) give an extensive coverage of the upstream and downstream of the sphere to illustrate the impact of Grashof number and position. The width of the temperature decreasing or the increase in velocity along the radial direction represents the thickness of the thermal boundary layer and the velocity boundary layer, respectively.

Figure 6 shows that all the variations in temperature decrease monotonically at various polar angles with increasing radial distance. However, as the fluid receives continuous heat from the sphere and flows downstream from the front stagnation point, its temperature rises, leading to a gradual reduction in heat transfer capacity. As a result, the surface temperature increases with increasing angle $\theta$; from this inference, the highest temperature is located at the rear stagnation point ($\theta =180^\circ$), which is also very good in accordance with the characteristics of the isothermal contours. When $\theta <120^\circ$, the temperature decreases steeply to zero within a small distance from the surface of the sphere. At angles greater than 120° ($\theta >120^\circ$), the temperature decline is more gradual, attributed to the thickening of the thermal boundary layer as it develops. Comparing Fig. 6a with Fig. 6b, it can be found that the temperature decays to zero at a radial distance of approximately three times the sphere radius for $Gr=10$, whereas it decreases to zero when ${R}^{*}\approx 1.7$ at $Gr={10}^{7}$. This evidences the boundary layer’s thinning with a rising Grashof number, corroborating the predictions of boundary layer theory.

Figures 7 and 8 show the distributions of the tangential velocity and radial velocity profiles along the radial directions at different angles. In Fig. 7, all the tangential velocity profiles increase steeply to a maximum value and then decrease moderately with increasing radial distance except for $\theta =0^\circ$, which always remains zero, and the distributions of which are in good agreement with the velocity boundary layer. Moreover, when $0^\circ <\theta \le 120^\circ$, the highest values of the tangential velocity increase with increasing $\theta$. This suggests that the fluid surrounding the surface is accelerated as a result of the favorable pressure gradient. As the plume begins to form at $\theta >120^\circ$, the maximum values of the tangential velocity decrease afterwards for higher $\theta$ owing to the thickening of the boundary layer. In addition, the value of the tangential velocity for $Gr={10}^{7}$ is larger than that for $Gr=10$. It is interpreted that the buoyancy of the fluid strengthens, resulting in a weakening of the resistance with increasing Grashof number. As shown in Fig. 8, the values of radial velocity are negative for the sphere when the $\theta \le 120^\circ$ for $Gr=10$ and the $\theta \le 150^\circ$ for $Gr={10}^{7}$, which implies the inflow region where the fluid flows toward the sphere. When the angles come to high value, the direction of the flow changes, and the fluid moves away from the surface of the sphere. Hence, the radial velocity variations are positive for $\theta >120^\circ$ or $150^\circ$, which is regarded as the outlet region. With increasing of angles, the maximum value of the radial velocity shifts forward along the dimensionless radial distance. Moreover, the variations of the radial velocities tend to approach zero at the high Grashof number ($Gr={10}^{7}$). Comparing the radial velocity changes of $\theta =150^\circ$ and $\theta =165^\circ$ at $Gr={10}^{7}$, it can be seen that their curves have a large difference in value, which is due to the combined effects of the boundary layer development and the interaction with the sphere, leading to the thickening velocity boundary layer. In addition, this interaction of them weakens the velocity boundary layer on the top surface of the sphere at a larger angle, as seen in Fig. 5. On the other hand, due to the intensified inertia forces, the flow separation is delayed as the Grashof number varies from $10$ to ${10}^{7}$, leading to an additional reduction in the thickness of the velocity boundary layer at larger angles. That is the reason why there is a significant variation in the radial velocity between $\theta =150^\circ$ and $\theta =165^\circ$.

### Local drag coefficient distribution

**Figure 9:**
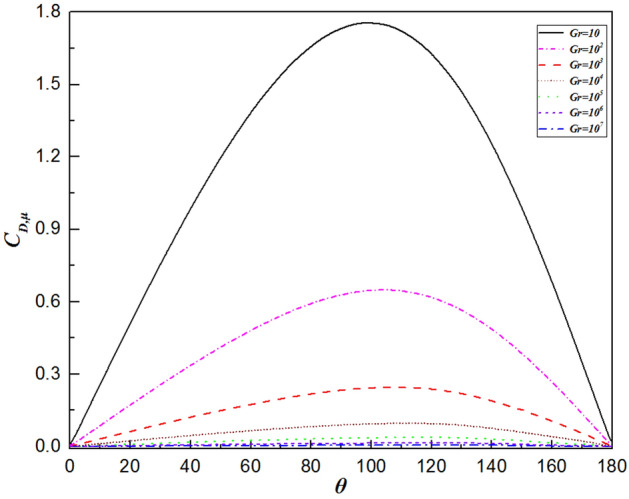
Variation in the local friction drag coefficient along the surface of the sphere for different Grashof number.

**Figure 10:**
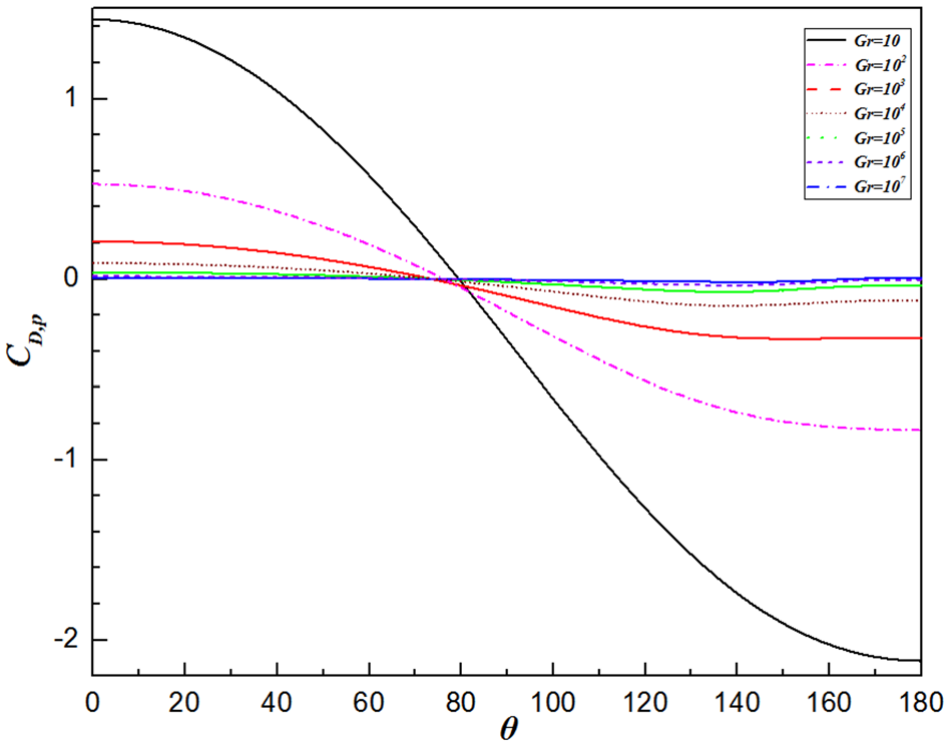
Variation in the local pressure drag coefficient along the surface of the sphere for different Grashof number.

The variation in the local drag coefficient (${C}_{D}$) represents the common influence of the various forces in the flow direction, comprising both the local friction drag coefficient (${C}_{D,\mu }$) and local pressure drag coefficient (${C}_{D,p}$), as defined in Eqs. (16) and (17), respectively. Therefore, changes in the local drag coefficient provide important insights into the fluid flow dynamics around a sphere. Overall, the local friction and pressure drag coefficients exhibit completely different trends depending on the Grashof number, as shown in Figs. 9 and 10, respectively. Analysis using Figs. 9 and 10 reveals that the local friction drag coefficient arises from changes in the velocity gradient on the sphere’s surface, ${(\partial {U}_{\theta }/\partial r)}_{wall}$, as shown in Eq. (16). The fluid is accelerated by the favorable pressure gradient presented in Fig. 10 from the front stagnation point $(\theta =0^\circ )$. The velocity gradient in the vicinity of the sphere surface increases, thereby leading to an increase in the local friction drag coefficient, as observed in Fig. 9. Then, the local friction drag coefficient reaches the maximum value at $\theta =98.6^\circ \sim 115.2^\circ$ depending upon the Grashof number. This is in good agreement with the velocity gradient on the surface of the sphere achieving the maximum value. Although the velocity of the free flow continues to increase due to the favorable pressure gradient when $\theta >98.6^\circ \sim 115.2^\circ$ in Fig. 10, the local friction drag coefficient decreases because of the thickening boundary layer owing to the formation of the plume. At the sphere’s apex, the fluid ceases to accelerate, impacted by the unchanged pressure gradient. Ultimately, both the fluid velocity and the local friction drag coefficient reduce to zero at the rear stagnation point. Moreover, as shown in Fig. 9, the maximum value moves downward along the surface of the sphere with increasing Grashof number, i.e., $\theta =98.6^\circ$ for $Gr=10$ and $\theta =115.2^\circ$ for $Gr={10}^{7}$. This is because the viscosity of the fluid decreases with increasing Gr. Similarly, the minimum point of the local pressure drag coefficient advances upstream as the Grashof number increases.

### Local Nusselt number distribution

**Figure 11:**
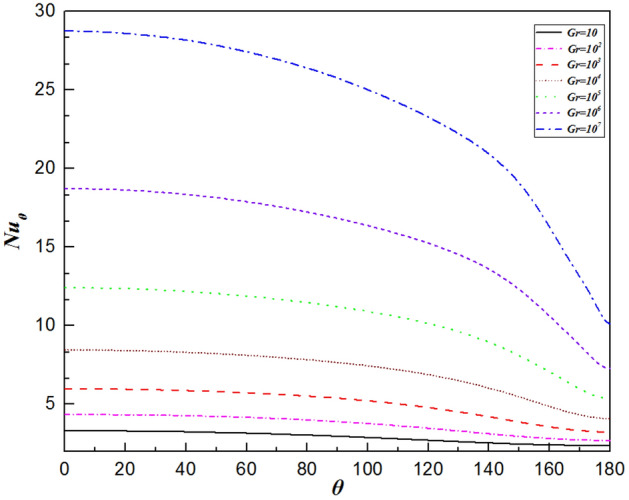
The variation in the local Nusselt number along the surface of the sphere for the Grashof number varied from $Gr=10$ to $Gr={10}^{7}$.

The local Nusselt number is determined by the temperature gradient perpendicular to the sphere’s surface. Moreover, the variation in the local Nusselt number along the sphere surface is also affected by the local velocity field. Regardless of the Grashof number, the local Nusselt number gradually decreases from the maximum value at the front stagnation point ($\theta =0^\circ$), as shown in Fig. 11. Conversely, the minimum Nusselt number is found at the rear stagnation point ($\theta =180^\circ$), where the thermal boundary layer thickens with increasing $\theta$. The following mechanisms are considered. Firstly, a reduced temperature difference along the sphere surface naturally leads to a decrease in the heat transfer rate. Furthermore, as previously noted, the velocity gradient continues to increase due to the decreasing pressure (shown in Fig. 10). Although an increase in the fluid velocity will lead to an increase in the heat transfer rate, the impact of the temperature difference accounts for a greater proportion of the increase, which does not reverse the decrease in the rate of heat transfer. Overall, the local Nusselt number rises with increasing Grashof number. A smaller *Gr* results in higher fluid viscosity. This is due to the weak buoyancy force, which decreases the flow velocity to the fluid. That is, the relative proportion of conductive heat transfer is greater than that of convection. In contrast, when the Grashof number is large, the thermal boundary layer becomes thinner, reducing the thermal resistance of conduction across the boundary layer. As a result, the variation in the values of the local Nusselt number is more extensive than that for smaller Grashof number.

Furthermore, for $\theta <128^\circ \sim 142^\circ$ (depending on the Grashof number), the slope of the variation in the local values of the Nusselt number decreases slowly (shown in Fig. 11). This slow decrease results from two competing effects: the boundary layer thickening and velocity increase due to the favorable pressure gradient. However, when $\theta >128^\circ \sim 142^\circ$, the curve drops steeply. This is because as the angle increases continuously, the temperature difference between the sphere surface and fluid decreases gradually. Moreover, the fluid cannot be accelerated due to the disappearance of the pressure gradient. These factors cause the heat transfer rate to decline rapidly.

### Comparison with an isothermal sphere

**Figure 12:**
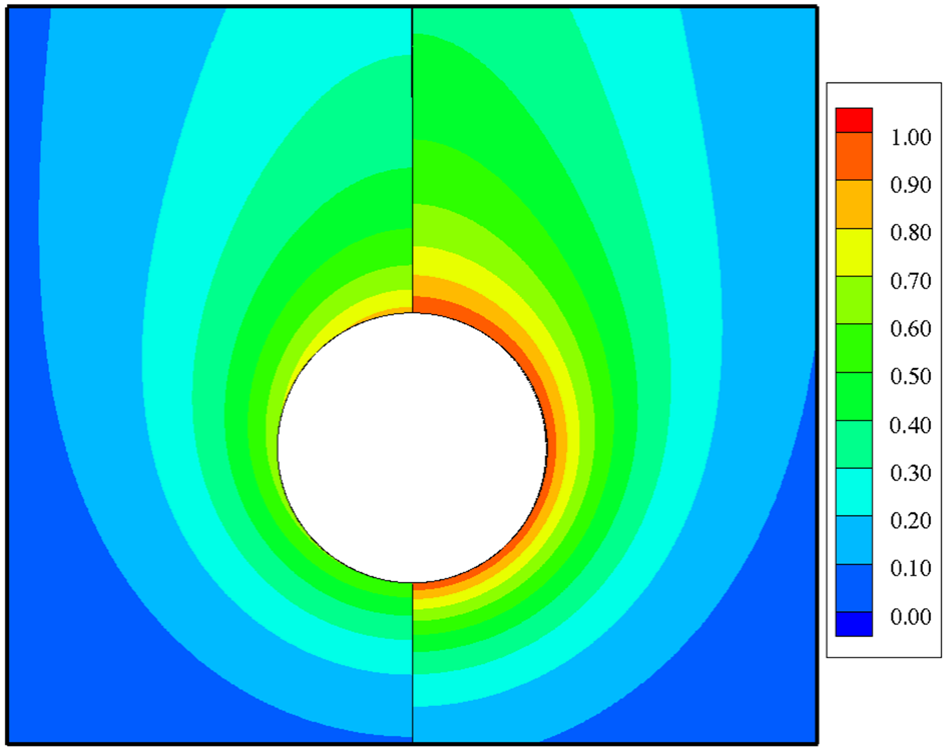
Isotherm contours for constant heat flux (left half) and constant temperature (right half) in the vicinity of the sphere for $Gr=10$.

**Figure 13:**
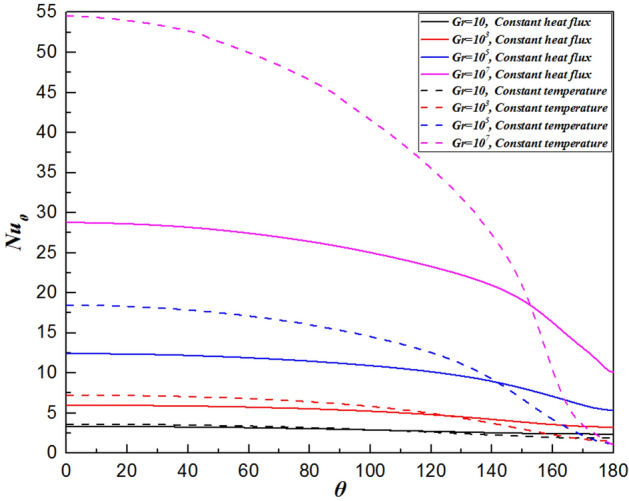
The variation in the local Nusselt number along the sphere surface for the Grashof number varied from $Gr=10$ to $Gr={10}^{7}$.

**Figure 14:**
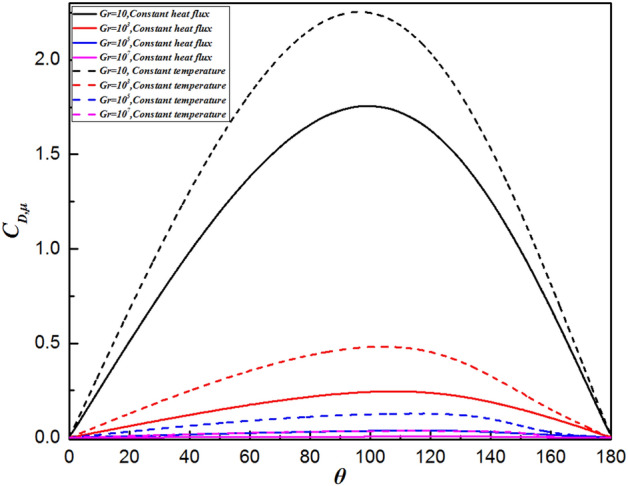
The variation in the local friction drag coefficient along the sphere surface for the Grashof number varied from $Gr=10$ to $Gr={10}^{7}$.

**Figure 15:**
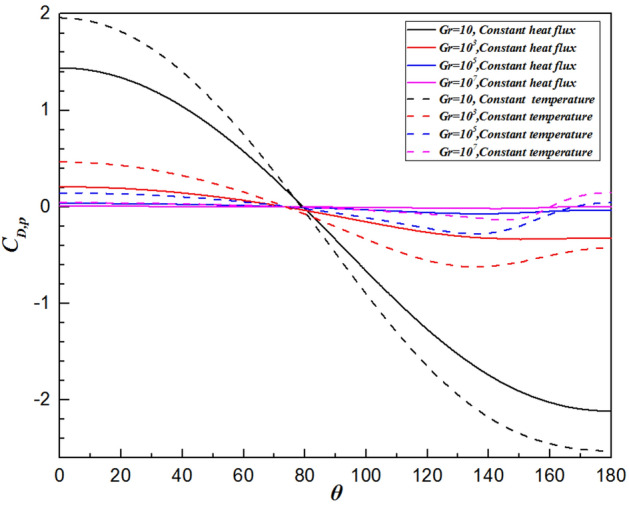
The variation in the local pressure drag coefficient along the sphere surface for the Grashof number varied from $Gr=10$ to $Gr={10}^{7}$.

Figure 12 shows the comparison of the isothermal contour at $Gr=10$, with the left half depicting a single sphere under constant heat flux and the right half an isothermal sphere. There is a significant difference in thermal boundary layer thickness, with the isothermal sphere exhibiting a larger temperature difference than the constant heat flux sphere at the same Grashof number. As a result, the differences in temperature fields will be explained by analyzing the distributions of ${Nu}_{\theta }$, ${C}_{D,\mu }$ and ${C}_{D,p}$. For natural convection heat transfer over a single sphere, the trends of the three parameters are almost identical whether the surface is subjected to a constant temperature or uniform heat flux. Generally, the sphere with a constant temperature surface has higher maximum values for the Nusselt number, friction drag coefficient, and pressure drag coefficient compared to the sphere with constant heat flux. The curve of the local Nusselt number for the isothermal sphere is steeper than that for the sphere with constant heat flux. However, for $\theta =112^\circ \sim 151^\circ$ (depending on the Grashof number), the value of the local Nusselt number for sphere with constant heat flux is larger than that for isothermal sphere, as shown in Fig. 13. This results from the combined effects of the velocity gradient and the temperature difference between the sphere’s surface and the adjacent fluid. The variation in the local pressure drag coefficient reflects the change in the fluid velocity gradient, as shown in Fig. 15. When $\theta >130^\circ$ for the isothermal sphere, the appearance of an adverse pressure gradient illustrates that the fluid is decelerated. In contrast, at large angles for the constant heat flux sphere, the local pressure drag coefficient curve is gentler, with the pressure gradient approaching zero. Similarly, at larger angles, the thermal boundary layer of the isothermal sphere becomes significantly thicker. These two effects determine that the heat transfer rate in the upper part of the sphere decreased drastically compared with that in the constant heat flux sphere. Moreover, the isothermal sphere provides a larger temperature gradient, resulting in the fluid flow providing a greater driving force, which causes an increase in the fluid velocity gradient near the surface of the sphere, leading to an increase in the local friction coefficient, as shown in Fig. 14. The larger the Grashof number is, the thinner the velocity boundary layer is. The local friction drag coefficient inversely correlates with the Grashof number. This underscores the significant impact of the velocity gradient on the heat transfer rate in natural convection over a single sphere (Fig. 15).

### Average Nusselt number

**Figure 16:**
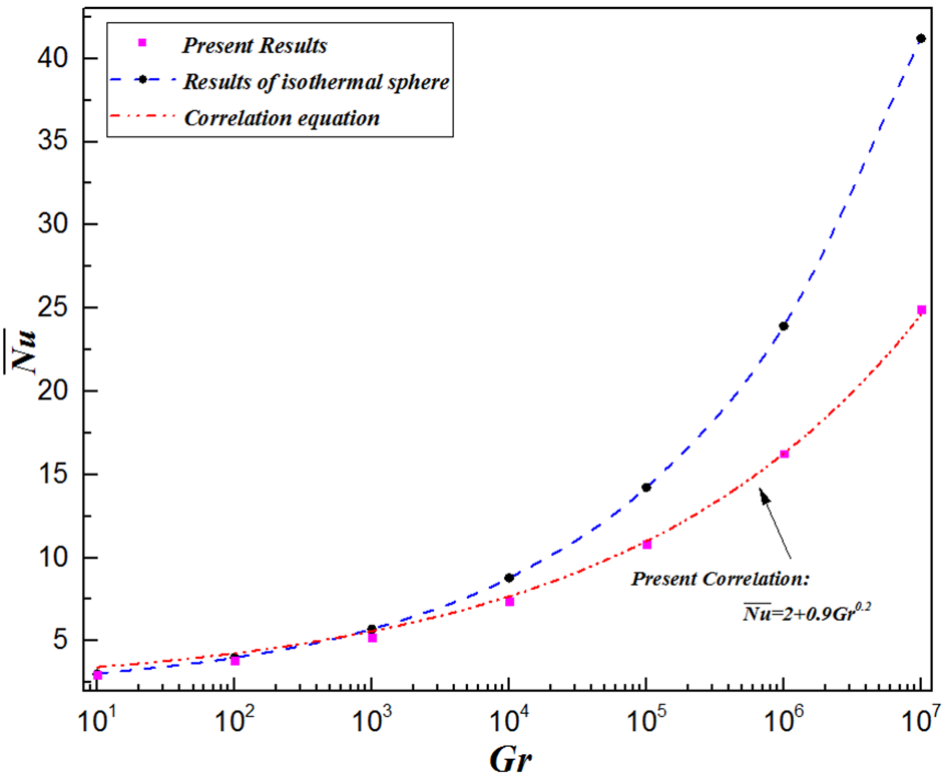
Correlation for the present average Nusselt number and comparison of the boundary condition of constant heat flux with that of constant temperature for a single sphere.

The average Nusselt number plays an important role in actual engineering applications because the average rate of natural convective heat transfer is generally considered in engineering calculations. Figure 16 shows the average Nusselt number ($\overline{Nu }$) as a function of the Grashof number for ${ }Pr = 0.72$. In Fig. 16, the red dots denote the numerical results of the average Nusselt number from the boundary condition of constant heat flux, while the red line represents the fitting curves derived from the numerical simulation. Figure 16 shows that the Nusselt number increases with increasing Grashof number, which can be attributed to the reinforcing convection resulting from the increase in the Grashof number. The results can be correlated to predict the heat transfer rate from a single sphere over the range of Grashof number ($10 \le Gr \le 10^{7}$) studied in this work, employing the following correlation:

19 $$\overline{Nu} = m + n \cdot Gr^{l}$$

For the heat transfer of the laminar natural convection of the sphere, the general form of the average Nusselt number ($\overline{Nu}$) is generally $2 + n \cdot Gr^{l}$, i.e., the parameter $m$ is set to 2. It is worth mentioning that “$\overline{Nu} = 2$” represents the limiting value of the Nusselt number corresponding to heat conduction without heat convection from the sphere set to a constant heat flux. It can be obtained by solving the conduction Eq. ^5^. This is consistent with the correlation equations in paper^10,20,39,40^. Then, according to the values of $m$, the values of the fitting parameters $n$ and $l$ are equal to $0.9$ and $0.2$, respectively. The Nusselt number in the present work varies as $Gr^{0.2}$, which is different from that of an isothermal sphere (varies as $Gr^{0.25}$) and shows a fundamental difference from the boundary conditions of constant heat flux and constant temperature at the surface of a single sphere^5^. Finally, the expression equation was ultimately obtained as follows:

20 $$\overline{Nu} = 2 + 0.9 \cdot Gr^{0.2}$$

The mean error associated with Eq. (20) is only 1.88%, which increases to the largest deviation of 9.02%. The determination coefficient of Eq. (20), $R^{2} = 0.997$, is such a high degree of precision that it guarantees consistency with the present numerical data. This correlation can be employed to accurately calculate the overall Nusselt number for natural convection from a constant heat flux sphere over a wide range of Grashof number ($10 \le Gr \le 10^{7}$) in air. Moreover, it can also provide more accurate predictions with verifiable results from relevant experiments.

On the other hand, the blue and red line show the fitting curves of the average Nusselt number over a single sphere compared with the boundary conditions of constant temperature and constant heat flux, respectively. The average Nusselt number for the isothermal sphere is almost equal to that of the constant heat flux sphere when $Gr \le 10^{3}$. However, the ratio difference between the two different boundary conditions gradually increases with increasing Gr number. A faster fluid velocity and a larger temperature gradient cause the isothermal sphere to achieve a greater overall heat transfer rate. Table 7 shows a comparison of the average Nusselt number for a single sphere submitted to a constant heat flux and isothermal surface based on the Grashof number. The Grashof number has a great impact on the average heat transfer. The ratio of the average heat transfer rate (${ }\overline{{Nu_{I} }} /\overline{{Nu_{F} }} { }$) is ${ }0.986$ for $Gr = 10$, but it is ${ }0.604$ for ${ }Gr = 10^{7}$. Therefore, it is inadvisable to predict the heat transfer rate for the case of a sphere submitted to constant heat flux using the correlating equations for an isothermal sphere.

**Table 7:** Variations in the ratio $\overline{{Nu_{I} }} /\overline{{Nu_{F} }}$ as $Gr$ for the average heat transfer.

$Gr$	$\overline{{Nu_{I} }}$	$\overline{{Nu_{F} }}$	$\overline{{Nu_{I} }} /\overline{{Nu_{F} }}$
$10$	2.981	2.939	0.986
${10}^{2}$	3.987	3.795	0.952
${10}^{3}$	5.705	5.195	0.911
${10}^{4}$	8.778	7.369	0.839
${10}^{5}$	14.218	10.808	0.760
${10}^{6}$	23.920	16.259	0.680
${10}^{7}$	41.212	24.904	0.604

## Conclusions

In the present study, laminar natural convection heat transfer over a single sphere subjected to constant heat flux in air was investigated numerically over the range of Grashof number $10 \le Gr \le 10^{7}$. The improved and accurate computational model proposed by Bejan et al.^28^ can significantly reduce the size of the computational domain. The extensive results, such as the streamline and isothermal contours, the local pressure and friction drag coefficients, the local and average Nusselt number distribution have been presented. Furthermore, the above results of the spherical surface maintained at a constant heat flux are compared with those at a constant temperature. The obtained conclusions can be found as follows:

1. By comparing with previous work, the feasibility and accuracy of the numerical and physical model used in this paper is verified adequately. The obtained results achieved for validating the domain and grid independence tests have been in good agreement these of Ref.^17^ and Ref.^23^ and have been given credibility to further study.
2. The heat transfer capacity of the sphere with constant heat flux is obviously much weaker than that of the isothermal sphere. The ratio of the average Nusselt number for the isothermal and constant heat flux sphere surface (${ }\overline{{Nu_{I} }} /\overline{{Nu_{F} }} { }$) is 0.986 for ${ }Gr = 10$, 0.911 for $Gr = 10^{3}$, 0.760 for $Gr = 10^{5}$, and 0.604 for $Gr = 10^{7}$, which is attributed to the isothermal sphere exhibits a stronger temperature gradient, and the difference of the temperature gradients between the spheres with two different surfaces become larger as the Grashof number increasing.
3. Due to the weakening of the shear stress, both the local friction coefficient and the pressure drag coefficient decrease with increasing Grashof number which represents that the total drag coefficient decreases with the increasing *Gr*. Furthermore, the maximum local friction drag coefficient shifts downstream with increasing Grashof number, which is caused by the variation of the boundary layer and pressure gradient along $\theta$. For example, the maximum value is $\theta = 98.6^\circ$ for $Gr = 10$, whereas it is $\theta = 110.6^\circ$ for ${ }Gr = 10^{5}$, while ${ }Gr = 10^{7}$, it shifts to ${ }\theta = 115.2^\circ$. Compared with the isothermal sphere, the local pressure drag coefficients for the constant heat flux do not show an adverse pressure gradient at large angles. That is due to the common effects of simultaneously decelerating the fluid velocity and thickening the temperature boundary layer leads to a dramatic reduction in the heat transfer rate.
4. The value of the local Nusselt number shows a positive dependence on the Grashof number and a negative dependence on the polar angle $\theta$, respectively. The effect of polar angle is mainly related to two factors: velocity gradient and temperature gradient. With the $\theta$ growing, the negative effect of the gradual decreasing of the temperature gradient appeared. However, the negative impact is moderated to the gentle slope by the initial positive velocity gradient from $\theta = 0^\circ$ to about $140^\circ$ for $Gr \ge 10^{3}$. After the $\theta$ is further increased, the velocity gradient plays a negative role. Due to enhanced convective heat transfer, this law becomes more evident at higher Grashof number. Moreover, when $\theta < 112^\circ \sim 151^\circ$ (depending on the Grashof number), the curve of the local Nusselt number for sphere with constant heat flux is lower than that for isothermal sphere. However, the opposite is true for $\theta > 112^\circ \sim 151^\circ$. For the isothermal single sphere surface, the heat transfer rate may slightly either enhance or lower relative to that for the single sphere with uniform heat flux surface, based on the two competitive influences of velocity gradient and temperature gradient.
5. The precise correlated equation of the average Nusselt number in $10 \le Gr \le 10^{7}$ is presented as follows:
  $$\overline{Nu} = 2 + 0.9 \cdot Gr^{0.2} .$$

The accuracy and reliability of the simulation results are highly important from both practical and academic points of view.

## Data Availability

The datasets generated during and/or analysed during the current study are available from the corresponding author on reasonable request.

## List of symbols

$C_{D}$: Drag coefficient (dimensionless)
$C_{D,\mu }$: Friction drag coefficient (dimensionless)
$C_{D,p}$: Pressure drag coefficient (dimensionless)
$D$: Diameter of sphere (m)
$D_{\infty }$: Distance of outer computational boundary (m)
$$g$$: Gravitational acceleration (m/s^2^)
$Gr$: Grashof number (dimensionless)
$h_{\theta }$: Local heat transfer coefficient (${\text{W}}/{\text{m}}^{2} \;{\text{K}}$)
$\overline{h}$: Average heat transfer coefficient (${\text{W}}/{\text{m}}^{2} \;{\text{K}}$)
$$k$$: Thermal conductivity (W/m K)
$n$: Power-law index (dimensionless)
$Nu$: Nusselt number (dimensionless)
$\overline{Nu}$: Average Nusselt number (dimensionless)
$p$: Gauge pressure (Pa)
$Pr$: Prandtl number (dimensionless)
$q_{s}$: Surface heat flux (${\text{W}}/{\text{m}}^{{2}}$)
$$R$$: Sphere radius (m)
$r$: Spherical radial coordinate
*Ra*: Rayleigh number (dimensionless)
$s$: Surface area of sphere (m^2^)
$T$: Temperature of fluid (K)
$T_{m}$: Film temperature of fluid (K)
$T_{\infty }$: Temperature of fluid far away from sphere (K)
$T_{w}$: Temperature of the surface of the sphere (K)
$u,{ }v$: $x$ And $y$ components of the velocity (m/s)
$$V_{\theta }$$: Tangential velocity (m/s)
$$V_{R}$$: Radial velocity (m/s)
$U,{ }V$: $x$ And $y$ components of the velocity (dimensionless)
$$u_{c}$$: Reference velocity (m/s)
$$x,{ }y$$: Cartesian coordinates (m)
$X,{ }Y$: Cartesian coordinates (dimensionless)

## Greek symbols

$\alpha$: Thermal diffusive coefficient (m^2^/s)
$\beta$: Coefficient of thermal expansion (K^−^^1^)
$$\vartheta$$: Kinematic viscosity (m^2^/s)
$$\varepsilon$$: Nondimensional temperature
$\rho$: Density of the fluid (kg/m^3^)
$\tau_{w}$: Shear stress of the sphere surface (Pa)

## Superscript and subscripts

$$*$$: Dimensionless quantities
$$\theta$$: Local value
$I$: Sphere with a isothermal surface
$F$: Sphere with a constant heat flux surface
